# Complementary Contribution of Wild Bumblebees and Managed Honeybee to the Pollination Niche of an Introduced Blueberry Crop

**DOI:** 10.3390/insects12070595

**Published:** 2021-06-30

**Authors:** Marcos Miñarro, Daniel García

**Affiliations:** 1 Servicio Regional de Investigación y Desarrollo Agroalimentario (SERIDA), Villaviciosa, 33300 Asturias, Spain; 2 Departamento de Biología de Organismos y Sistemas, Universidad de Oviedo and Unidad Mixta de Investigación en Biodiversidad (CSIC-Uo-PA), 33004 Asturias, Spain; danielgarcia@uniovi.es

**Keywords:** *Apis mellifera*, *Bombus pascuorum*, *Bombus pratorum*, *Bombus terrestris*, crop pollination, environmental niche, foraging behaviour, niche segregation, spatio-temporal segregation, *Vaccinium ashei*

## Abstract

**Simple Summary:**

Blueberry is a crop that is increasing globally, both in terms of yield and extent, mostly thanks to the recent introduction of North American blueberry in the temperate areas of Europe, Asia, and South America. As blueberry depends largely on insects for pollination, farmers in these expansion areas face the challenge of adapting this crop to unexpected pollinator species, whose traits and features may not entirely fit the pollination needs of the introduced crop. Here we study the abundance, the behaviour, and the response to environmental conditions of different managed and wild native pollinators of blueberry in northern Spain. Our findings suggest the dominant role of native wild bumblebees and managed honeybee as suppliers of pollination service. Honeybee and bumblebees differed in both when and where they occurred, in how they responded to environmental conditions, and in how they behaved as an effective pollinator. The role of bumblebees and honeybee as blueberry pollinators thus seems complementary and additive. These results encourage the preservation of populations of native wild bees in order to ensure the effective management of introduced blueberry crops.

**Abstract:**

The entomophilous pollination niche (abundance, phenotypic traits, foraging behaviours and environmental tolerances of insect pollinators) helps to understand and better manage crop pollination. We apply this niche approach to assess how an entomophilous crop (blueberry, *Vaccinium ashei*) can be expanded into new territories (i.e., northern Spain) far from their original area of domestication (North America). Insect visits to blueberry flowers were monitored in a plantation on 12 different days, at 8 different times during day and covering various weather conditions. Abundance, visitation rate, pollen gathering behaviour, and frequency of inter-plant and inter-row movements were recorded. The pollinator assemblage was basically composed of one managed honeybee species (50.8% of visits) and three native bumblebee species (48.3%). There was a marked pattern of seasonal segregation throughout bloom, with bumblebees dominating the early bloom and honeybee the late bloom. Pollinators also segregated along gradients of daily temperature and relative humidity. Finally, the two pollinator types differed in foraging behaviour, with bumblebees having a visitation rate double that of honeybee, collecting pollen more frequently and changing plant and row more frequently. The spatio-temporal and functional complementarity between honeybee and bumblebees suggested here encourages the consideration of an integrated crop pollination strategy for blueberries, based on the concurrence of both wild and managed bees.

## 1. Introduction

The production of many crop plants around the world depends greatly on pollination by insects, including both managed (e.g., honeybee) and wild (e.g., bees, hoverflies, butterflies) species [1,2,3]. One concept for understanding the role of insects for different crops is the pollination niche: the multidimensional array of insect species features (abundance, phenotypic traits, foraging behaviours, and tolerance to abiotic conditions) that determine the pollination success of each plant species [4]. On the one hand, the pollination niche helps to understand the response of crop yields to environmental change. For example, the loss of seminatural habitats in agricultural landscapes and the concomitant decline of wild pollinators of crops may compromise yields as managed honeybees alone cannot compensate for the lost effectiveness of wild pollinators [3,5]. This is because of the positive effect of pollinator richness, which derives from the functional complementarity of the different insect species [6,7]. On the other hand, a pollination-niche approach also seems necessary for estimating how entomophilous crops can be expanded into new territories far from their original areas of domestication [8]. In this sense, little is known about how introduced crops must necessarily rely on farmer input (through managed bees) or may benefit from the pre-adapted action of native wild pollinators (see [9]).

Blueberry (*Vaccinium* spp. Ericaceae) as a crop has shown a marked increase in global yield, as well as a strong expansion worldwide [10]. This geographic expansion mostly entails the recent introduction of North American species (e.g., *V. corymbosum*, *V. ashei,* etc) into temperate areas of Europe, Asia, and South America [10]. In these new areas, farmers face the challenge of adapting this crop to unexpected pollinator assemblages given that blueberry crops are highly dependent on insect pollination [11,12,13]. For example, fruit set in blueberry flowers with free access to insects was found to be 14.5 times higher than in flowers from which pollinators were excluded [12]. In their original distribution range, blueberry crop is pollinated by highly diverse communities of wild bees [14,15,16], although honeybee hives are frequently used to complement pollination by wild insects [12,16,17]. Nevertheless, knowledge of blueberry pollinator assemblages outside North America is scarce and fragmentary (e.g., [18] in Estonia; [19] in Chile; [13] in Australia) and there is an urgent need for studies to determine how complementary the different pollinator types (managed vs. wild species) are. In this sense, complementarity may emerge from the segregation of the different pollinators in time, space, and environmental conditions, as well as from the functional differences associated with foraging behaviour [7,20,21].

Blueberry crops have long bloom periods (more than four weeks) that frequently extend beyond the short lifespan of many solitary bees. Thus, it could be expected that different bee species visit blueberry flowers in different moments throughout the bloom period, although gregarious pollinators, such as honeybees and bumblebees (both wild and managed) have, as a colony, longer life periods. However, their phenology as blueberry pollinators may be constrained by factors such as the time or the location of hive settlement [22] or floral competition from wild plants or from other crops [23,24,25]. Negative interactions between honeybees and wild bees when accessing flowers may also occur [26,27,28]. We can therefore expect phenological and spatial (e.g., between individual plants) segregation of pollinators during the bloom period, with different bee species providing pollinator services to blueberry in different periods and to different individual plants [29,30,31,32]. Moreover, and due to the long bloom period and the relatively long-lived flowers, blueberry pollinators must cope with wide gradients of temperature and relative humidity conditions within single days or between different days [33]. In this sense, different tolerances to abiotic conditions are expected among species, due, for example, to thermoregulation ability (with bumblebees being able to fly under lower temperatures and higher humidity; [34]). Finally, the specific contributions of particular insect species to blueberry pollination would also be related to differences in their foraging behaviour and movement, leading to different pollen loads and qualities [20,35,36]. Due to blueberry flower traits (narrow-opening bell-shaped corolla, protected poricidal anthers, protruding stigma; [37,38]) large differences in pollen load are expected between species (e.g., due to buzzing behaviour, bumblebees—but not honeybees—are able to buzz and release pollen from the poricidal anthers of blueberry flowers, [39,40] but see [41]).

Blueberry crops are rapidly expanding in Spain, which has in fact become the top producer in Europe (53,380 t in 2019) and the fourth in the world [42]. Although most of the yield is obtained in the south, under a Mediterranean climate, the crop in northern Spain, under an Atlantic climate, is also increasing and it is of great strategic interest because harvesting is in summer when southern production has ended. In the north, local farmers currently rely on honeybee hives and commercial bumblebee colonies at varied densities to ensure blueberry pollination [43]. However, no specific pollination studies have been conducted in the area to prove that managed pollination is required and to determine the role of native pollinator communities. Here, we apply a niche approach to understand the entomophilous pollination of blueberry crops introduced into northern Spain. By differentiating between native wild bees (i.e., bumblebees) and managed honeybee, we sought to answer the following questions: (1) Do honeybee and native bumblebees segregate as pollinators across space and time?; (2) Do these pollinator types show overlapping or different tolerances to abiotic conditions?; (3) Are there differences between pollinators in foraging behaviour?

## 2. Materials and Methods

### 2.1. Study Site

The study was carried out during the blueberry bloom period in a commercial plantation located in Villaviciosa (Asturias), (43°30′46″ N, 5°20′38″ W; 20 m above sea level). Asturias has a temperate oceanic climate with rainfall usually exceeding 1100 mm, which is fairly evenly spread out over the year. The plantation, 13.6 ha in size, was planted in 2008 with several cultivars of both *V. corymbosum* and *V. ashei*. The study was conducted in a plot of 1 ha of *V. ashei* cv. Ochlockonee with cv. Powderblue as the pollinizer planted in the same rows at a ratio of 1:10. The plantation was surrounded mainly by eucalyptus plantations (ca. 60% cover in a 1000 m radius), pastures, shrubs, and apple crops. The plantation groundcover was mechanically mowed several times and during the sampling there were almost no flowers on the ground. Three hives of the European honeybee (*Apis mellifera*) (were settled in the plantation margin before the bloom started. One commercial hive (three colonies) of the bumblebee *Bombus terrestris* (Tripol, Koppert Biological Systems, Berkel en Rodenrijs, Netherlands) was introduced on April 15th when the bloom had already started. No pesticides were used during the pollinator observations.

### 2.2. Pollinator Monitoring

Pollination of blueberry by insects was monitored on 12 different days between late March and early May (28, 30 March; 1, 5, 6, 14, 19, 22, 29 April; 3, 5, 9 May) 2016 at eight different times of day (from 10:00 h to 17:00 h). To address certain questions (see ‘Environmental niche of bumblebees and honeybee’ in Section 2.5, Statistical analysis), we distinguished a posteriori two study phases of similar duration: early bloom (28/03–14/04/2016; with six monitoring days) and late bloom (19/04–9/05/2016; with six monitoring days). The distinction was based on strong differences in flowering phenology (Figure S1), pollinator assemblage composition (see Section 3.1, Relationship between bumblebee and honeybee abundance), and average temperature (see Section 2.3, Environmental conditions).

At the beginning of the bloom, 20 blueberry plants from 6 rows in the centre of the plot were randomly selected and marked. In each of the 96 censuses (12 dates x 8 times), one observer stood for 1 min in front of each of the 20 selected plants, counting and visually identifying all pollinators (i.e., insects visiting blueberry flowers), which were classified as European honeybee, bumblebees (several species), wild bees, hoverflies, or flies. On each sampling day, for each selected plant we counted the number of flowers open, still closed or already fallen, and calculated the corresponding percentages for open, closed, and fallen flowers by averaging data from the 20 selected plants every sampling day (Figure S1).

### 2.3. Environmental Conditions

For each census, we registered temperature and relative humidity data from a portable weather station (PCE-AM 82, PCE Instruments) placed among the plant rows and protected from direct sunlight. The long bloom period of blueberry (almost 1.5 months in our study) enabled a wide variety of weather conditions to be covered during censuses (temperature: mean ± SD 19.6 °C ± 4.6, min–max 11.6–33.4 °C; relative humidity: mean ± SD 46.9% ± 14.2, min-max 18.9–77.5%). Temperature and relative humidity were negatively correlated (r = −0.765; N = 96; *p* < 0.001). Temperature average and dispersion was significantly higher in late bloom (21.7 °C ± 4.7 SD) than in early bloom (17.5 °C ± 3.4 SD) but relative humidity was similar between bloom periods (Table S1).

### 2.4. Foraging Behaviour of Pollinators

We studied the foraging behaviour of honeybee and bumblebees in terms of four different functional parameters: frequency of pollen gathering, the number of flowers visited per minute (visitation rate), the frequency of inter-plant movements and the frequency of inter-row movements (how often a pollinator left an individual plant to visit another, and whether the new plant was in a different row). *Vaccinium ashei* cultivars are partially self-sterile and thus, the movement of pollinators between plants and crop rows is important to secure pollen transfer from pollinizer plants to crop plants [38,44].

Pollen gathering was registered during the monitoring of pollinators in censuses (see above) by recording the presence of pollen in the corbiculae of each individual pollinator. Blueberry flowers have poricidal anthers, meaning pollen is not freely accessible but restricted to insects capable of vibrating the anthers, i.e., buzz-pollinating insects [39]. Consequently, insects capable of gathering pollen from blueberry flowers are expected to be better pollinators of this crop [39].

Visitation rate and insect movements between plants and rows were sampled in the study site in additional observations independent of census monitoring. To do this, any flower visitor detected during a slow walk along plant rows was visually tracked until lost, recording tracking time, number of flowers visited, and whether each visited flower was on the same or another plant and in the same or another plant row.

### 2.5. Statistical Analysis

#### 2.5.1. Relationship between Bumblebee and Honeybee Abundance

We sought to evaluate the relationship between the abundances of bumblebees (all *Bombus* species pooled) and honeybee visiting flowers across censuses by taking into account the potential statistical pseudoreplication derived from the fact that different censuses were conducted on the same date, time, or plant. For this, we built a Generalized Linear Mixed Model (GLMM) using, as a response variable, the number of bumblebees observed per census, considering a zero-inflated Poisson error distribution and a log-link function. As fixed-effect predictors we considered the number of honeybees per census (main effect) and the number of open flowers per plant per census (covariable). The model also incorporated date of observation (with 12 levels), time of observation (with 8 levels) and plant identity (with 20 levels) as random-effect factors.

#### 2.5.2. Environmental Niche of Bumblebees and Honeybee

We represented the environmental niche of bumblebees and honeybee from the response of these different pollinators to the environmental space shaped by the gradients of temperature and relative humidity across the study period. To do so, we first used GLMMs to estimate the response of the abundances of bumblebees and honeybee (as response variables in different models) to temperature and relative humidity (as fixed predictors). Different error distribution families were chosen for the different models, depending on the structure of the response variables (Gaussian, Poisson, or zero-inflated Poisson; Table S2), and date and time of census as random factors (data from different plants were pooled for each time and date). Different models were developed for early and late bloom periods (Table S2). Second, and separately for early and late bloom, we estimated the predicted values of these models as quantitative estimates of the response of the different pollinators to the same environmental space. We inferred overlap or segregation of environmental niche from the sign and the degree of significance of the correlation between the responses of bumblebees and honeybee, again, separately for early and late bloom.

#### 2.5.3. Foraging Behaviour Differences between Pollinators

We compared the different functional parameters of foraging behaviour between pollinator species (honeybee and three bumblebee species). We used different GLMMs with the functional parameters as response variables, considering different error distribution families depending on the structure of these parameters: binomial for pollen gathering frequency and inter-row movement frequency, Gaussian for visitation rate, and Poisson for inter-plant movement frequency. All models considered species identity as fixed main predictor, and date and time of observation as random effects. Multiple comparisons of means between species were made a posteriori by means of Tukey contrasts.

## 3. Results

### 3.1. Relationship between Bumblebee and Honeybee Abundance

We recorded 5657 insect visits to blueberry flowers. All the observed visits were legitimate, that is, no robbing behaviour was recorded (e.g., Rogers et al., 2013a). Honeybees and bumblebees were the dominant pollinators, accounting for 50.8% and 48.3% of total number of observed individuals, respectively. Different dipterans (mainly hoverflies) (0.8%) and solitary bees (0.1%) completed the assemblage of floral visitors. We recorded three species of bumblebees, *Bombus terrestris* being the dominant species (83.9% of bumblebee observations), followed by *B. pratorum* (9.5%), and *B. pascuorum* (6.6%).

**Figure 1:**
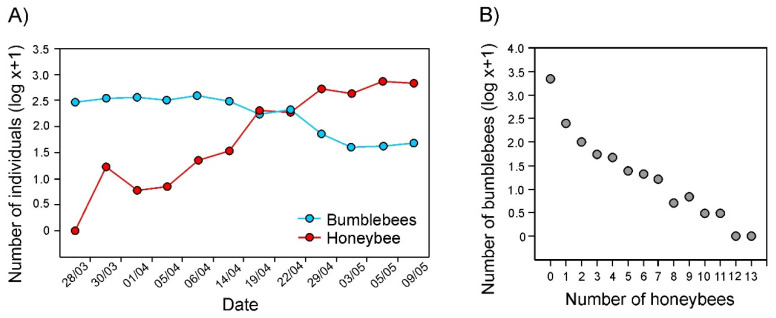
(**A**) Variation in the number of bumblebee (*Bombus* spp.) and honeybee (*Apis mellifera*) individuals in censuses (data from different times and plants pooled) on different dates throughout the sampling period. (**B**) The cumulative number of bumblebee individuals observed in censuses with different numbers of honeybees observed (data from different dates pooled).

Despite observing similar overall numbers of individual bumblebees (2731) and honeybees (2872), the distribution of abundance of the two pollinators strongly differed across the sampling period. Bumblebees were the dominant pollinator in the first half of the bloom period, whereas they decreased dramatically in the number later in the bloom period, when honeybees dominated the assemblage (Figure 1A). Nevertheless, irrespective of the date and the time of census, the number of bumblebees observed per census was negatively related to the number of honeybees, suggesting that the different insects visited different plants when they co-occurred in the plantation (Figure 1B; Table 1). No effect of open flower abundance was found on the number of bumblebees per census, when the effects of date and time on census data were considered (Table 1).

The three bumblebee species followed a similar pattern of abundance across the study period, being higher in early bloom but decreasing progressively in late bloom (Figure S2A). Hence the abundances of the three species were positively correlated (r > 0.622; *p* < 0.05, in all paired comparisons; Figure S2B).

### 3.2. Environmental Niche of Bumblebees and Honeybee

**Figure 2:**
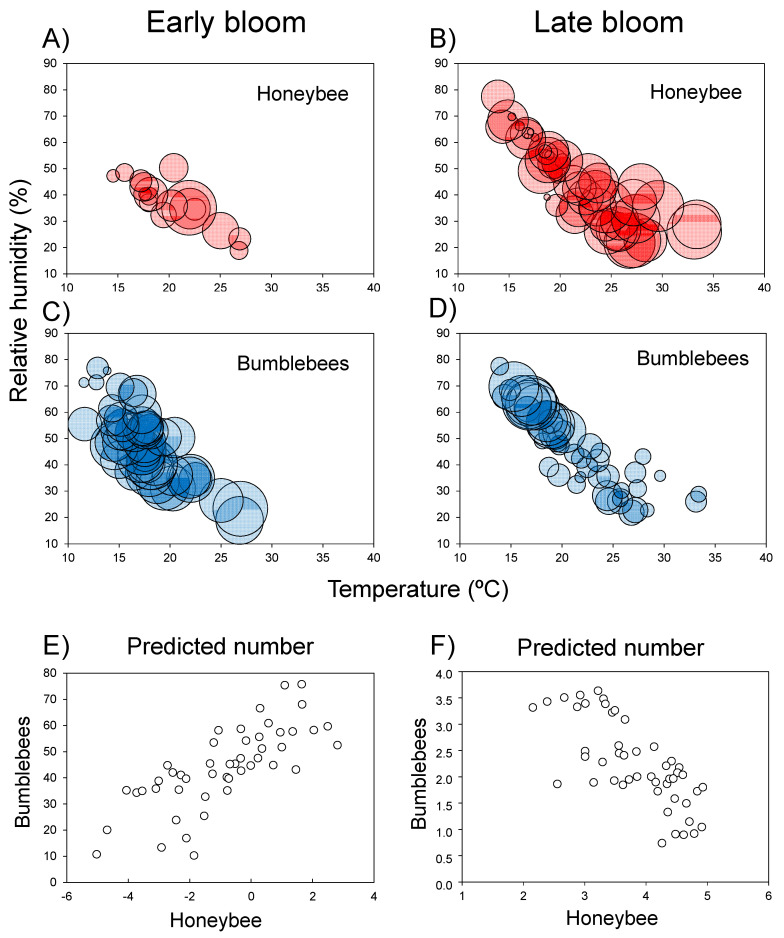
Abundance of honeybees (*Apis mellifera*) and bumblebees (*Bombus* spp.) visiting blueberry flowers according to temperature and relative humidity conditions in early and late bloom (**A**–**D**). Relationships between the predicted numbers of honeybees and bumblebees in early and late bloom (**E**,**F**). The bubble size in (**A**–**D**) reflects the magnitude of the abundance.

During the early bloom period, the few honeybees recorded during censuses visited blueberry flowers mainly under the restricted conditions of medium-to-high temperatures (17–25 °C) and relatively low humidity values (25–45%; Figure 2A). Bumblebees occurred throughout the complete gradients of environmental conditions, both in temperature (including below 15°C and above 25 °C) and in relative humidity, although they were more abundant under lower relative humidity (Figure 2C; Table S2). As judged from the predicted values of pollinator abundance in relation to temperature and humidity, environmental tolerances of bumblebees and honeybee were positively correlated during early bloom (Figure 2E).

During late bloom, honeybee was distributed across the complete range of temperature and humidity, but was more abundant on census days and at times with higher temperature and lower humidity (Figure 2B). However, bumblebees mostly appeared under low temperature and high humidity and only occurred occasionally in the inverse environmental conditions (Figure 2D). Therefore, in contrast to during early bloom, the environmental tolerance of bumblebees and honeybee were negatively correlated during late bloom (Figure 2F).

Similar patterns of time-dependent discordance of environmental tolerance between bumblebees and honeybee were interpretable from the distribution of abundances in relation to temperature and time of day (Figure S3).

### 3.3. Foraging Behaviour Differences between Pollinators

**Figure 3:**
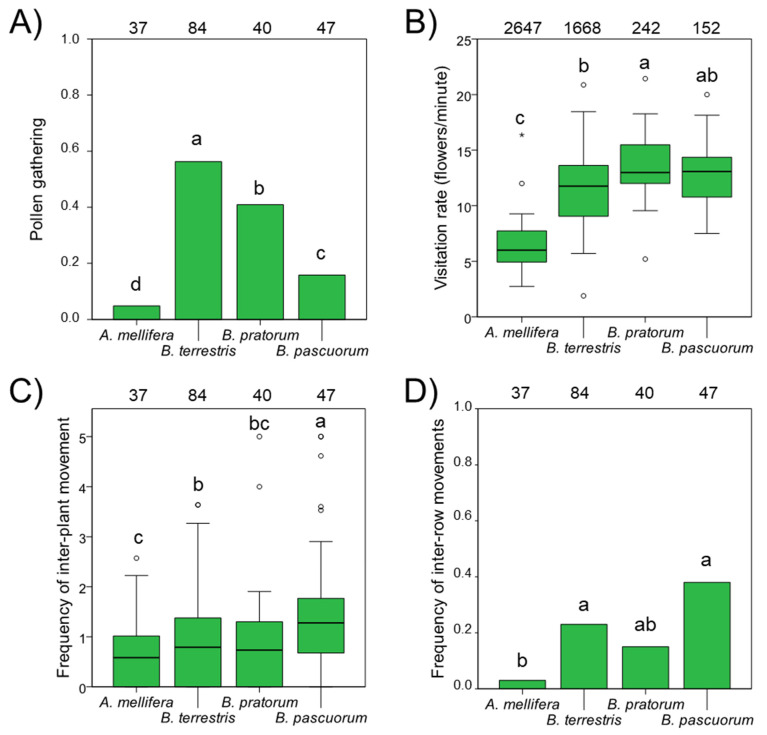
Pollen gathering (frequency of individuals with pollen in their corbiculae) (**A**), visitation rate (number of blueberry flowers visited per minute) (**B**), frequency of movements between plants (**C**) and between plant rows (**D**) for each pollinator species. Numbers at the top of each column indicate sample size. For each plot, different letters indicate differences between pollinator species. For (**B**,**C**), boxplots indicate 25–75% quartiles (box boundaries), median (thick horizontal bar), largest and smallest observed values (whiskers), outliers (small circles), and extreme values (asterisks). For each plot, different letters indicate differences between pollinator groups.

Honeybee differed from bumblebees in all functional parameters of pollinator foraging behaviour. All bumblebee species showed a higher frequency of pollen gathering than honeybee (Table 2; Figure 3A). Among bumblebees, *B. terrestris* collected pollen more frequently (56.2% of individuals) than *B. pratorum* (40.9%) and *B. pascuorum* (15.8%) (Figure 3A).

Pollinator species differed significantly in their flower visitation rate (flowers/min), with bumblebee rates being double that of honeybee (6.4 flowers/min) (Table 2; Figure 3B). Among bumblebees, *B. pratorum* visited more flowers per minute than *B.* *terrestris* (Figure 3B).

Honeybee also showed a significantly lower frequency of movements between plants than *B. pascuorum* and *B. terrestris* (Table 2, Figure 3C) as well as moving between rows less frequently than these two bumblebee species (Table 2) (Figure 3D).

## 4. Discussion

In this study, we assessed the pollination niche of blueberry, a recently introduced crop, in northern Spain. Our findings confirm the dominant role of native wild bumblebees and managed honeybee as suppliers of pollination service. Relative to honeybee, wild bumblebees showed marked segregation in spatio-temporal occurrence and environmental tolerances to abiotic conditions, as well as differences in foraging behaviours affecting effectiveness as pollinators. The relative contribution of bumblebees and honeybee to the pollination niche of blueberry thus seems to be complementary and additive. These results encourage the preservation of populations of native wild bees in order to ensure the effective management of introduced blueberry crops.

### 4.1. Spatio-Temporal Segregation in the Blueberry Pollinator Assemblage

The pollinator assemblage was composed of honeybee, three bumblebee species, and a few anecdotal solitary bees and dipterans (less than 1% of visits). This represents a reduced pollinator assemblage compared to that of blueberry crop species in their native range (e.g., [16,30,45]), as well as with those of other native and exotic fruit crops in the study region (e.g., apple [20]; kiwifruit [46]). Such a low number of pollinator species seems likely to be more related to biological reasons such as, for example, floral morphology and biology (bell shape, narrow opening, enclosed poricidal anthers; [38]), rather than to sampling constraints (ca. 5700 observations across 96 20-min censuses) or a lack of diversity in local native pollinator communities. Concerning their provenance, the honeybees most likely came from hives installed in the plantation studied or others in the surrounding areas. *Bombus pascuorum* and *B. pratorum* are native, non-commercial species and thus they would have come from wild populations. *Bombus terrestris*, the most common bumblebee in this study (and in fruit crops in the region, [20,46]), is a native but also commercial species. In fact, a *B. terrestris* hive was introduced in the plantation but only at the beginning of the late bloom period, when visits by this pollinator decreased. Thus, we can assume that the vast majority of *B. terrestris* visits observed here were made by wild populations. In sum, wild populations of native bumblebees contributed greatly to the pollination of this introduced blueberry, in accord with findings from other studies outside North America [18,19].

Bumblebees and honeybee visited blueberry flowers at practically the same total frequency (48.3% and 50.8%, respectively). However, the relative frequency of these two pollinator types changed dramatically over time, namely a massive increase in honeybee during late bloom. There was, therefore, a pattern of very clear temporal segregation of flower visitation between bumblebees and honeybee. Different co-occurring mechanisms, such as variation in blueberry and alternative floral sources and resource-related interspecific competition, may underpin this temporal segregation. On the one hand, there was a positive correlation between bumblebee abundance and the average number of open flowers per census date (r = 0.856; *p* < 0.001, N = 12 dates). Then, the progressive decrease of bumblebees in blueberry over the bloom period could be related with the lower availability of blueberry flowers as the sampling period proceeded (Figure S1). On the other hand, the virtual absence of honeybee in early bloom coincided with the massive flowering of *Eucalyptus globulus*, a timber plantation species abundant in the landscape surrounding the plantation studied (ca. 60% of area in a 1000 m radius). Compared to the morphologically restrictive blueberry, the eucalyptus flower represents a very accessible and abundant source of pollen and nectar for generalist anthophagous insects such as honeybee [47,48]. We hypothesize, then, that honeybee concentrated on eucalyptus during blueberry early bloom but, as eucalyptus flowering decreased, they spilled over into blueberry crops. Finally, the spatial repulsion between bumblebees and honeybee when they co-occur in the plantation, inferred from the time-independent and significant negative effect of honeybee abundance on bumblebee abundance, suggesting some kind of interspecific competitive interaction [26,27].

### 4.2. Environmental Segregation between Bumblebees and Honeybee

Besides the temporal segregation across the bloom period, we also found evidence of environmental segregation between bumblebees and honeybee along the gradients of temperature and relative humidity, at least in late bloom. In early bloom, the few honeybees present visited blueberry flowers mainly in the mornings, and preferentially on hot dry days, disappearing from the crop under other conditions, whereas bumblebees occurred in all conditions, though also responding positively to increased temperature. The narrow environmental tolerance of honeybee was, therefore, nested within the wider niche of bumblebees. In late bloom, honeybee occurred preferentially in the morning or even in the afternoon when temperatures were high, but never on cold afternoons when, contrastingly, bumblebees occurred the most (Figure S3). The two pollinator types, in this way, complemented each other to supply the pollination service across a wide space of environmental conditions. This environmental segregation mostly reflected the liberation of flower resources for bumblebees under conditions of worse weather, most likely enabled by their greater capacity to remain active at low temperatures [33,49].

### 4.3. Differences in Foraging Behaviour between Bumblebees and Honeybee

Bumblebee species and honeybee showed marked differences in terms of foraging behaviour. These differences were independent of the date and time when observations were made, suggesting that they are the consequence of specific traits. First, all bumblebees collected pollen more frequently than honeybee, the latter mostly being observed to just gather nectar. Bumblebees, but not honeybee, are able to buzz and release pollen from the poricidal anthers of blueberry flowers [39]. As a consequence, the probability of covering their bodies with pollen and exchanging it between flowers in order for effective pollination to occur is higher in bumblebees than in honeybee [39,40]. Second, the three bumblebee species visited twice as many flowers as honeybee, confirming that bumblebees are faster pollinators of blueberry plants than honeybee [39,40]. Third, both *B. pascuorum* and *B. terrestris* moved between blueberry plants and between rows more frequently than honeybee, a behaviour that aids with pollen exchange between blueberry cultivars [11]. In sum, as judged from the foraging behaviour, and relative to the honeybee, we may expect bumblebees to provide an increased magnitude and quality of pollination service to blueberry crops [36,39,40,50].

### 4.4. Complementarity between Bumblebees and Honeybee and the Pollination Niche of Blueberry

There is increasing evidence that more diverse assemblages of flower-visiting insects provide better pollination services and increase yields (through increased fruit-set and seed-set) in several crops (e.g., [51,52]), including blueberry [30,53]. Functional complementarity, i.e., the fact that different species complement each other in functional terms, is one of the mechanisms widely used to explain the positive yield effects of richer pollinator assemblages [51,54]. Among the sources of complementarity are the segregation of pollination activity across time and space (i.e., pollinators differ in the date or the time of the day they visit the crop or in the area of the crop they visit; [54,55,56], the segregation of responses to environmental gradients (i.e., different responses lead to ensuring pollination service irrespective of environmental variability; [20,33,57,58], and divergence in functional traits and behaviours (i.e., phenotypic variability across species leads to qualitative differences in pollination which may compensate for differences in abundance of pollinators; [30,52,54]. Our results evidence all three of these sources of complementarity, suggesting that the current combined contribution of wild native bumblebees and managed honeybee to pollination niche entails a higher magnitude and stability of fruit crop of introduced blueberry in northern Spain.

## 5. Conclusions

This study evidences that American blueberry crops introduced into northern Spain show traits that facilitate high enough levels of flower visitation by insects that yields comparable to those found in the native area can be achieved (e.g., [43]. Farmers can, therefore, be confident of achieving effective levels of pollination service by managing honeybee and relying on the spontaneous and, remarkably, free activity of wild native bumblebees. The suggested complementarity between bumblebees and honeybee should be profitable for farmers not only as a result of additivity but also through the stabilizing effects of securing visits to blueberry flowers at different times and under various meteorological conditions. Consequently, as a practical recommendation, a scheme of integrated crop pollination strategy (sensu [59]), based on the concurrence of populations of both wild and managed bees, should be promoted. To promote populations of wild bumblebees, local farmers are encouraged to apply ecological intensification measures, such as sowing flowers at the farm scale [60]) or decreasing farm intensity at the landscape level [45,61]. However, new research to understand how this complementarity between honeybee and bumblebees translates into the pollination service of blueberry crops is welcome, given the high dependence of this crop on insect pollinators [12].

Whether farmers in northern Spain might decline to set up honeybee hives and rely exclusively on wild pollinators for blueberry pollination requires further analysis (see also [17]. Nonetheless, several facts suggest the potential sufficiency of bumblebees to complete the pollination niche of introduced blueberry. First, they represent high-quality pollinators for this plant species (this study; [36,39,50]. Second, they seem to show population sizes high enough for pollination, tracking the variable number of open flowers throughout the bloom (Figure S1). Third, they maintain their activity across different weather conditions, even during low temperatures and high humidity (Figure 2C). Fourth, we cannot reject the hypothesis of a negative interaction between bumblebees and honeybee, suggesting that bumblebees could most likely recover their displaced pollinator role in the absence of honeybee. Raising farmers’ awareness of insect biodiversity, through focusing on wild bumblebees, thus appears to be a major target for the sustainable farming of introduced blueberry in northern Spain.

## Figures and Tables

**Table 1:** Generalized linear mixed model evaluating the effects of the number of honeybees, *Apis mellifera,* per census and the number of open flowers per tree (fixed effect estimates ± SE) on the number of bumblebees, *Bombus* spp., per census. The error distribution family, the link function, the number of observations (N), as well as the variance (±SD) for date, time, and plant identity (random factors) are also shown.

Bumblebees (Zero-Inflated Poisson, log, N = 1920)			
	Estimate ± SE/SD	z	*p*
Honeybees	−0.105 ± 0.023	−4.559	0.0001
Flowers	0.001 ± 0.001	1.034	0.301
Date	0.547 ± 0.739		
Time	0.028 ± 0.168		
Plant	0.036 ± 0.190		

**Table 2:** Generalized linear mixed models evaluating the effect of species identity (*Apis mellifera* (taken as base level for comparison) *Bombus pascuorum*, *Bombus pratorum*, and *Bombus terrestris*) (fixed effect estimates ± SE) on different features of pollination behaviour. The error distribution family, the link function, the number of observations (N), as well as the variance (±SD) for date and time (random factors) are also shown.

**Pollen Gathering Frequency (Binomial, logit, N = 342)**			
	**Estimate ± SE/SD**	**z**	***p***
*B. pascuorum*	1.709 ± 0.260	6.55	0.0001
*B. pratorum*	3.027 ± 0.194	15.59	0.0001
*B. terrestris*	3.701 ± 0.162	22.77	0.0001
Date	0.203 ± 0.451		
Time	0.047 ± 0.218		
**Visitation Rate (flowers/min) (Gaussian, Identity, N = 208)**			
	**Estimate ± SE/SD**	**t**	***p***
*B. pascuorum*	6.054 ± 0.676	8.94	0.0001
*B. pratorum*	7.222 ± 0.681	10.59	0.0001
*B. terrestris*	5.407 ± 0.655	8.26	0.0001
Date	2.133 ± 1.460		
Time	2.492 ± 1.579		
**Inter-Plant Movement Frequency (Poisson, log, N = 208)**			
	**Estimate ± SE/SD**	**z**	***p***
*B. pascuorum*	0.840 ± 0.196	4.29	0.0001
*B. pratorum*	0.258 ± 0.221	1.16	0.2433
*B. terrestris*	0.461 ± 0.191	2.41	0.0152
Date	0.000 ± 0.000		
Time	0.001 ± 0.001		
**Inter-Row Movement Frequency (Binomial, logit, N = 208)**			
	**Estimate ± SE/SD**	**z**	***p***
*B. pascuorum*	3.107 ± 1.057	2.94	0.0032
*B. pratorum*	1.849 ± 1.106	1.67	0.0944
*B. terrestris*	2.354 ± 1.047	2.25	0.0253
Date	0.000 ± 0.000		
Time	0.001 ± 0.001		

## Data Availability

Data available on request from the corresponding author.

## Supplementary Materials

The following are available online at https://www.mdpi.com/article/10.3390/insects12070595/s1. Figure S1: Blueberry flowering phenology throughout the sampling period, showing percentage of still-closed, open, and already-fallen flowers. On each sampling day, we counted the number of flowers open, still closed or already fallen in 20 selected plants, and calculated the corresponding percentages by averaging data from the 20 plants every sampling day. Figure S2: Relative abundance of the three species of bumblebees visiting blueberry flowers throughout the sampling period (A), and correlation between the daily abundance of the three bumblebee species (B) (r = 0.742; *p* < 0.01 for *B. terrestris-B. pratorum*, and r = 0.622; *p* < 0.05 for *B. terrestris-B. pascuorum*; and r = 0.781; *p* < 0.01 for *B. terrestris-B. pascuorum*). Table S1: Generalized linear mixed models evaluating the difference in temperature and relative humidity between bloom periods (early vs. late). The error distribution family, the link type, the number of observations (N), as well as the variance (±SD) for date and time (random factors) are also shown. Models estimated with the lmer function from lme4 R-package. Table S2: Generalized linear mixed models evaluating the effect of temperature and relative humidity on the number of honeybees, *Apis mellifera,* and bumblebees, *Bombus* spp., per census, in early and late bloom. The error distribution family, the link type, the number of observations (N), as well as the variance (±SD) depending on date and time (random factors) are also shown. Figure S3: Response surface of honeybee and bumblebees visiting blueberry flowers according to temperature and time of day in early and late bloom. Dots within the plots indicate the combinations of time and temperature registered during sampling (censuses). Colour contours are interpolated from the number of visits recorded in censuses. The colour scales represent the number of visits. Response surfaces are generated using Surfer 8 (Golden Software Inc., Golden, CO, USA) through the interpolation of all time-temperature combinations recorded during all censuses (96) to show the expected values of visits in other conditions. They therefore represents the activity pattern of pollinators in response to time and temperature not only under the observed conditions but also under hypothetical ones, although this extrapolation should be considered more cautiously the further the hypothetical conditions are from those observed.
