# Supplementary material for: Complementary Contribution of Wild Bumblebees and Managed Honeybee to the Pollination Niche of an Introduced Blueberry Crop
Source: Insects. 2021 Jun 30;12(7):595. doi: 10.3390/insects12070595 (PMC8307808; doi:10.3390/insects12070595)
Supplement: Supplementary file 1 [file insects-12-00595-s001.zip › insects-1240908-supplementary.pdf]

Insects (2021)

Supplemental material

Complementary contribution of wild bumblebees and managed honeybee to the pollination niche of an introduced blueberry crop.

Marcos Miñarro, Daniel García

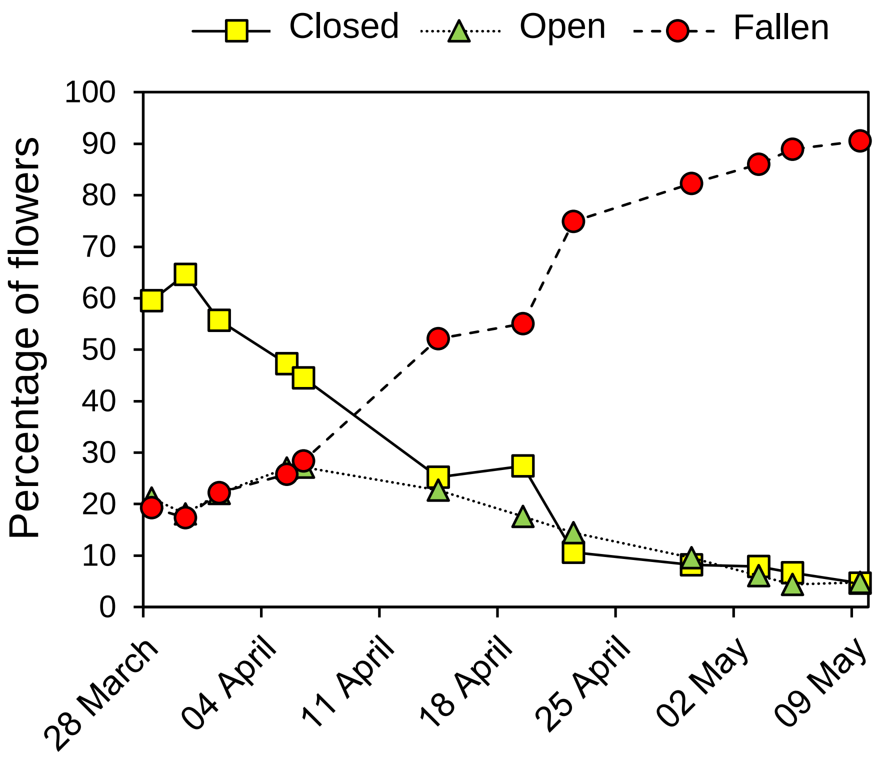

Figure S1. Blueberry flowering phenology throughout the sampling period, showing percentage of still-closed, open and already-fallen flowers. On each sampling day, we counted the number of flowers open, still closed or already fallen in 20 selected plants, and calculated the corresponding percentages by averaging data from the 20 plants every sampling day.

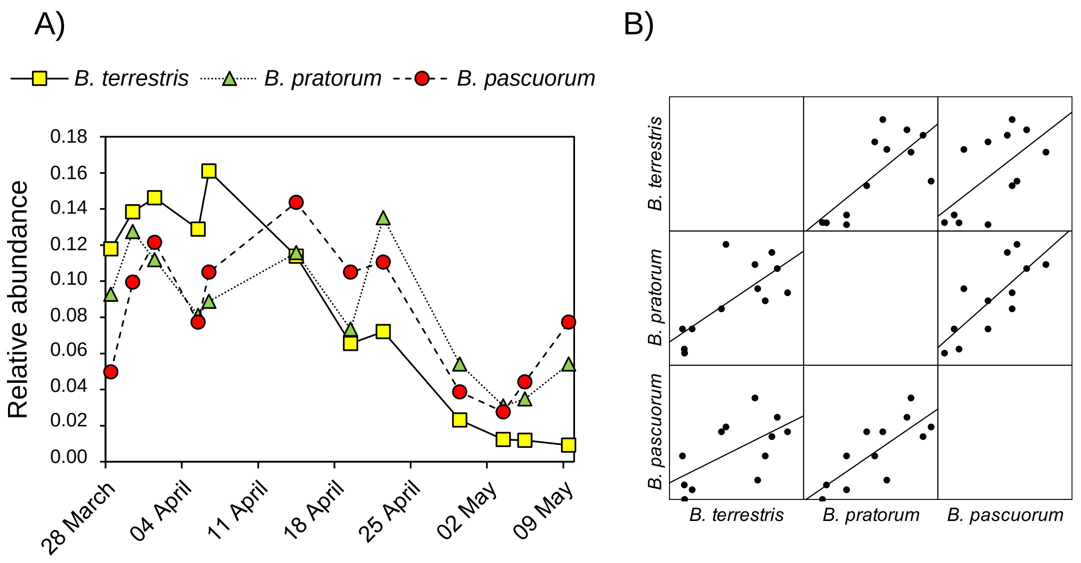

Figure S2. Relative abundance of the three species of bumblebee visiting blueberry flowers throughout the sampling period (A), and correlation between the daily abundance of the three bumblebee species (B) (r = 0.742; P < 0.01 for *B. terrestris - B. pratorum*, and r = 0.622; P < 0.05 for *B. terrestris - B. pascuorum*; and r = 0.781; P < 0.01 for *B. terrestris - B. pascuorum*).

Table S1. Generalized Linear Mixed Models evaluating the difference in temperature and relative humidity between bloom periods (early vs. late). The error distribution family, the link type, the number of observations (N), as well as the variance (±SD) for date and time (random factors) are also shown. Models estimated with lmer function from lme4 R-package.

**Temperature** (Gaussian, identity, N = 96)			
	Estimate ± SE/SD	t	P
Period (late)	4.154 ± 1.852	2.24	0.048
Date	9.444 ± 3.073		
Time	2.563 ± 1.601		
**Relative humidity** (Gaussian, identity, N = 96)			
	Estimate ± SE/SD	t	P
Period (early)	-2.913 ± 6.420	-0.45	0.659
Date	112.00 ± 10.58		
Time	15.31 ± 3.91		

Table S2. Generalized Linear Mixed Models evaluating the effect of temperature and relative humidity on the number of honeybee *Apis mellifera* and bumblebees *Bombus* spp. per census, in early and late bloom. The error distribution family, the link type, the number of observations (N), as well as the variance (±SD) depending on date and time (random factors) are also shown.

**Honeybee, early bloom** (Zero-inflated Poisson, log, N = 48)			
	Estimate ± SE/SD	z	P
Temperature	0.093 ± 0.155	0.59	0.5496
Relative humidity	-0.096 ± 0.040	-2.38	0.0171
Date	1.289 ± 1.136		
Time	0.554 ± 0.745		
**Bumblebees, early bloom** (Gaussian, identity, N = 48)			
	Estimate ± SE/SD	t	P
Temperature	-0.096 ± 0.886	-0.11	0.9267
Relative humidity	-1.197 ± 0.212	-5.64	0.0014
Date	3.999 ± 2.000		
Time	25.180 ± 5.018		
**Honeybee, late bloom** (Poisson, log, N = 48)			
	Estimate ± SE/SD	z	P
Temperature	-0.054 ± 0.017	-3.07	0.0022
Relative humidity	-0.048 ± 0.007	-6.77	0.0001
Date	0.198 ± 0.445		
Time	0.186 ± 0.432		
**Bumblebees, late bloom** (Poisson, log, N = 48)			
	Estimate ± SE/SD	z	P
Temperature	0.016 ± 0.045	0.35	0.725
Relative humidity	0.009 ± 0.018	0.52	0.605
Date	0.423 ± 0.650		
Time	0.222 ± 0.471		

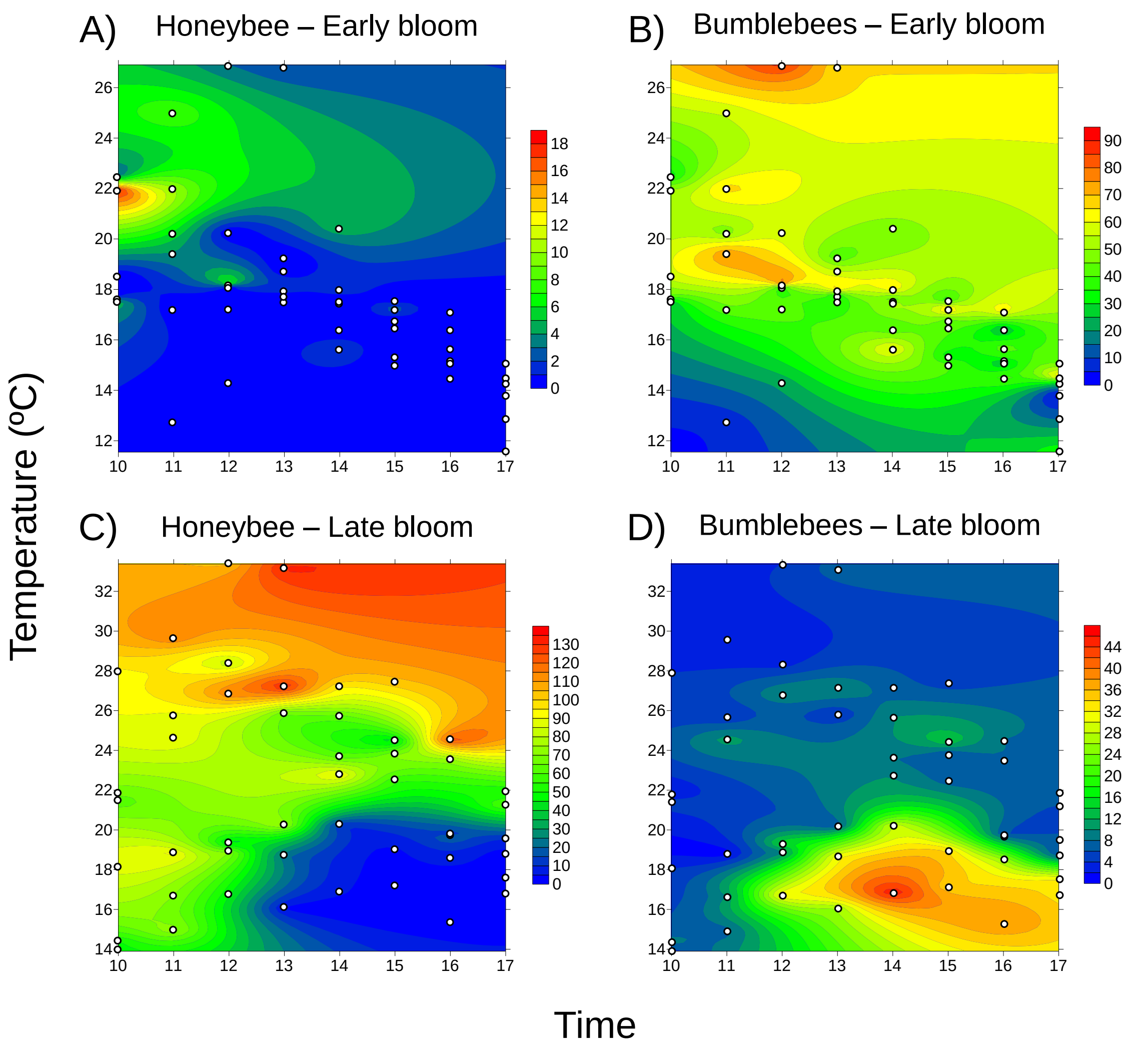

Figure S3. Response surface of honeybee and bumblebees visiting blueberry flowers according to temperature and time of day in early and late bloom. Dots within the plots indicate the combinations of time and temperature registered during sampling (censuses). Colour contours are interpolated from the number of visits recorded in censuses. The colour scales represent the number of visits. Response surfaces are generated using Surfer 8 (Golden Software Inc.) through the interpolation of all time-temperature combinations recorded during all censuses (96) to show the expected values of visits in other conditions. They therefore represent the activity pattern of pollinators in response to time and temperature not only under the observed conditions but also under hypothetical ones, although this extrapolation should be considered more cautiously the further the hypothetical conditions are from those observed.
